# Impact of kombucha, coffee, and turmeric beverages on the color stability of a single-shade versus a multi-shade resin-based composite

**DOI:** 10.7717/peerj.19759

**Published:** 2025-07-23

**Authors:** Hanin E. Yeslam, Abdulaziz F. Bakhsh

**Affiliations:** 1 Department of Restorative Dentistry, Faculty of Dentistry, King AbdulAziz University, Jeddah, Saudi Arabia; 2 Advanced Technology Dental Research Laboratory, King AbdulAziz Unniversity, Jeddah, Saudi Arabia; 3 Faculty of Dentistry, King AbdulAziz University, Jeddah, Saudi Arabia

**Keywords:** Color change, Composite resin, Single-shade composite, Esthetics, Turmeric, Restorative dentistry, Probiotic drinks, Esthetics, Staining, Nanofilled composite

## Abstract

Currently, patients place great significance on both the appearance and health benefits of dental treatments. Single-shade resin-based composites (RBC) are commonly used in clinical practice, but various popular caffeinated and healthy drinks can lead to staining. This study aimed to compare the resistance of a newly introduced single-shade RBC with an advanced polymerization system (APS) to turmeric, kombucha, and coffee staining as compared to that of a conventional multi-shade RBC. Sixty specimens (8 × 5 × 2 mm) were created using two types of RBCs: a single-shade (Vittra APS Unique, FGM Dental Group, Joinville, SC, Brazil) and a conventional universal RBC (Tetric N-Ceram, Ivoclar Vivadent, Germany). The samples were randomly divided into two main groups (*N* = 30) based on the RBC used. Each group was further divided into three subgroups (*n* = 10) based on the storage media, including turmeric, kombucha, and coffee (positive control). Color readings were recorded after 0, 6, and 12 days of immersion. The mean color change (ΔE (both ΔE*_ab_ and ΔE_00_)) values were statistically analyzed using repeated measure mixed design ANOVA and Bonferroni multiple comparisons (*p* < 0.05). The results of the study indicated that time had a statistically significant effect on ΔE (*p* < 0.001). The three-way interaction (time*material*solution) was also found to be statistically significant (*p* = 0.005). Turmeric caused a significantly higher ΔE compared to both coffee and kombucha (*p* < 0.001). Tetric N-Ceram exhibited a significantly lower ΔE than Vittra APS Unique (*p* = 0.004). In conclusion, with consideration of the limitations of the study, it can be stated that single-shade RBCs with APS are less color-stable than universal RBCs. The study also indicates that both kombucha and coffee can affect the color stability of RBCs, whereas turmeric has the most detrimental effect on the color of dental composites.

## Introduction

Patients currently visit dentists to improve the appearance of their teeth *via* direct and indirect restorations (Gray et al., 2021). Patients often worry about whether restorations will match and enhance their teeth’s color and shape. The success of esthetic dental restorative treatments is highly dependent on the proper and durable matching of shade to that of the adjacent tooth structures and other esthetic restorations (Piccoli et al., 2019), while evaluating their effect on the final tooth color (AlSheikh, 2019). Resin-based composite (RBC) materials are commonly used in esthetic restorations. Successful dental treatment using RBCs relies on the material’s mechanical and esthetic properties and its ability to withstand the oral environment over time (Bahbishi et al., 2020; Gray et al., 2021; Suneelkumar et al., 2021; Yeslam & AlZahrani, 2022). Since the 1950s, RBCs have evolved to offer a wide variety of opacities, shades, photo-initiators, and viscosities (Alhamed, Massoud & Doumani, 2021). Nanotechnology in dentistry has led to the development of RBCs featuring single-shade systems. These materials offer a color adjustment potential (CAP) that matches with the surrounding teeth, eliminates the need for complex shade-matching, and reduces material waste and inventory (Rohym, Tawfeek & Kamh, 2023). The distribution of spherical nano-filler particles with an average diameter of approximately 260 nm determines the ability of colors to blend and shift based on the angle of the light (Barros et al., 2023a). Additionally, using more transparent photo-initiators, such as the advanced polymerization system (APS) used in the recently introduced single-shade RBC (Vittra APS Unique, FGM Dental Group, Joinville, SC, Brazil), could improve translucency (Ismail & Paravina, 2022). Restorative materials face various challenges within the oral cavity that can potentially affect their color (Alhamed, Massoud & Doumani, 2021; AlSheikh, 2019). The discoloration of teeth and RBCs can result from two causes, namely, intrinsic or extrinsic. Intrinsic discoloration originates from within the teeth or RBCs, while extrinsic discoloration results from external factors such as smoking, as well as the consumption of certain foods and drinks (Bahbishi et al., 2020). The internal discoloration of RBCs over time is typically caused by the absorption of intra-oral stains. This process is influenced by the polymeric matrix, type of fillers, concentration, and photo-initiator system (Malekipour et al., 2012). The type of photoinitiator system can affect the color stability of RBCs. For example, RBCs based on Lucirin-2,4,6-trimethylbenzoyldiphenyl phosphine oxide (TPO) (*e.g.*, Tetric N-Ceram from Ivoclar Vivadent, Schaan, Liechtenstein) may exhibit better color stability than materials based on camphorquinone (CQ)/amine (Albuquerque et al., 2013; De Oliveira et al., 2015). The *in vitro* testing of restorative materials under various staining conditions could provide dental practitioners with a better understanding of the susceptibility of different restorative dental materials to discoloration (AlSheikh, 2019; Yeslam & AlZahrani, 2022). The staining medium influences the discoloration of RBCs, showing that different foods and drinks affect an RBC’s color uniquely (Ertan, Turkyilmaz & Ozkan, 2020; Gamal, Safwat & Abdou, 2022; Hasanain, 2022; Quek et al., 2018). In previous research, green and black teas, pigments found in turmeric and tomato sauce, cola drinks, and black coffee caused noticeable color changes in both single-shade and other nano-filled RBCs (Assaf, Abou Samra & Nahas, 2020; Ebaya et al., 2022; Gamal, Safwat & Abdou, 2022; Geethanjali et al., 2024; Hasanain, 2022). The staining effect of coffee and tea solutions was more noticeable on single-shade RBCs in some recent studies (Ahmed, Jouhar & Vohra, 2022; Ersöz et al., 2022). Universal micro-hybrid RBCs were found to have better color stability than other nano-filled RBCs when exposed to tea solutions (Malhotra et al., 2011). However, a recent randomized clinical trial found that single-shade color stability is clinically acceptable compared to a multi-shade Lucirin-TPO-based RBC (Anwar, Hussein & Riad, 2024). This supports the findings of previous *in-vitro* and *in-vivo* studies (Zulekha et al., 2022; Ebaya et al., 2022). According to a previous study, tea might cause a more noticeable color change in RBCs than coffee (Bahbishi et al., 2020). Kombucha beverages, essentially fermented teas, are becoming widely popular in today’s culture due to their probiotic and antimicrobial advantages (Nyiew, Kwong & Yow, 2022). It is important to note that these beverages have the potential to impact the surface roughness of dental restorations (Mert Eren et al., 2022). Hence, RBCs could be more susceptible to discoloration when exposed to tea. Turmeric consumption has become popular worldwide in recent years, expanding beyond its traditional use in ethnic cuisines. It is now being added to dairy- and plant-based milk, as well as fruit juices, to make use of its high nutritional value and its antimicrobial, antioxidant, and anti-inflammatory properties (Idowu-Adebayo, Fogliano & Linnemann, 2022; Sun et al., 2023). Turmeric solutions can significantly impact the color stability of various types of dental restorative materials, more so than tea, tamarind, other ethnic spices, and tobacco (Chittem, Sajjan & Varma Kanumuri, 2017; Telang et al., 2018; Thaliyadeth et al., 2019; Usha, Rao & George, 2018). These solutions contain a hydrophobic phytopigment, curcumin, which has the potential to cause yellowish discoloration in RBCs (Geethanjali et al., 2024). The deleterious effects of ingested beverages such as coffee, tea, turmeric, and red wine were also observed in gingiva-colored RBCs. However, drinks containing turmeric had the greatest impact on the RBC’s color stability (Marchan et al., 2020). Coffee contains caffeine, which can cause adverse health effects in high doses, such as nervousness, gastrointestinal disturbances, and cardiovascular symptoms (Nawrot et al., 2003). However, moderate coffee consumption up to three cups per day was reportedly associated with a reduction in metabolic disease risk and an increased antioxidant potential (Van Dam, Hu & Willett, 2020). Tannins, chlorogenic acids, melanoidins, and chromogens that are found in black coffee were linked to discoloration of dental hard tissues and restorations; however, the exact mechanism and dosage link remains controversial (Kim et al, 2025).

Many studies have examined the discoloration of composite resin restorative materials caused by popular beverages like coffee and tea. However, it is equally important to investigate the effects of beverages currently consumed by the modern population on these materials. Understanding their impact on the color stability of new and commonly used RBCs is crucial to ensure the durability and esthetic appeal of restorative dental work. Unfortunately, there is limited evidence in the literature regarding the impacts of commonly consumed beverages with probiotic, antioxidant, and caffeine contents on the color stability of newly developed single-shade dental resin-based composites (RBCs). Furthermore, no previous study has assessed the effects of probiotic kombucha drinks on the color stability of dental RBCs. The primary objective of this study was to investigate the effects of three popular beverages that are marketed for their health benefits—turmeric drink, green tea kombucha, and black coffee—at different exposure durations on the color stability of two different types of nano-hybrid resin-based composites (RBCs): a newly introduced single-shade RBC with APS and a universal multi-shade RBC with Lucirin-TPO. The first null hypothesis of the study was that there was no significant difference in the color change of the newly introduced APS-containing single-shade *vs.* a Lucirin-TPO-containing multi-shade RBC after exposure to the turmeric drink, green tea kombucha, and black coffee staining beverage solutions. The second null hypothesis was that there was no significant difference in the effects of these three beverages and/or the six, twelve, and eighteen-day exposure durations on the color stabilities of both tested RBCs.

## Materials and Methods

The study was conducted after gaining the approval of the ethical review committee of the Faculty of Dentistry at King Abdulaziz University, Jeddah, Saudi Arabia (IRB# 184-12-22).

### Study design

**Figure 1:**
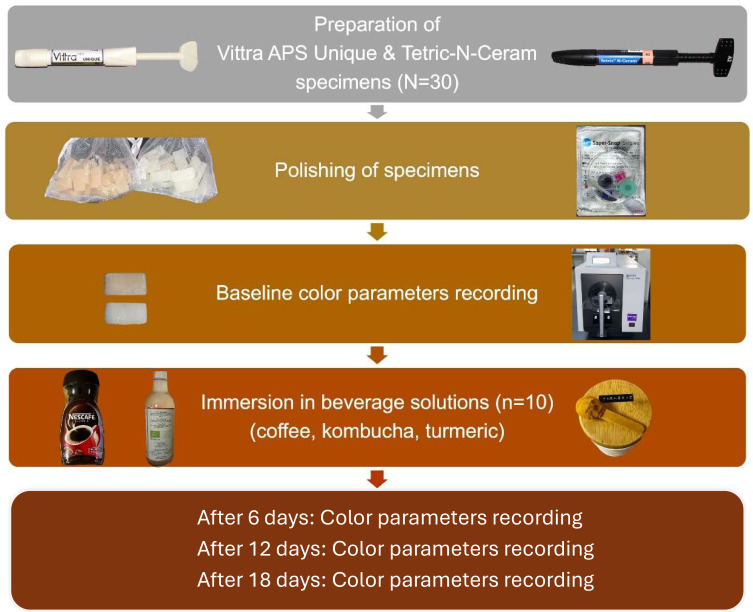
Diagram representing the study design of this *in vitro* study.

In this *in vitro* study, a total of 60 bar-shaped specimens, each measuring eight mm in length, five mm in width, and two mm in thickness, were produced. According to the sample size calculation conducted using the G*power software (G*power 3.1.9.7, Franz Faul, Universität Kiel, Kiel, Germany), a total sample size of 60 would provide a power of at least 0.90 with an effect size of 0.25 at a significance level of 0.05. The specimens were fabricated from two different RBC materials (*N* = 30 per material). The first was a recently introduced single-shade RBC with an advanced polymerization system (Vittra APS Unique, FGM Dental Group, Joinville, SC, Brazil), and the second was a commonly used shade A2 universal nano-hybrid RBC with Lucerin-TPO photoinitiator (Tetric N-Ceram, Ivoclar Vivadent, Schaan, Liechtenstein). Figure 1 summarizes the design of the current study. Vittra APS Unique (single-shade) and Tetric N-Ceram (multi-shade) were chosen to compare the newly introduced RBC with structural color adaptation to universal pigmented shade layering RBC, while controlling for filler load (∼65% Ba-glass), and a standardized irradiance (1,200 mW/cm^2^) and duration (20 s).

Each material group was divided into three subgroups based on the immersion beverage solution (*n* = 10); turmeric, kombucha, and coffee. The values of the color parameters L*, a*, and b* were measured according to the Commission Internationale de l’Eclairage (CIE) LAB color space, which expresses color in terms of lightness (L*), red/green intensity (a*), and yellow/blue intensity (b*) (Luo, 2014). The recorded color values were measured for all samples after finishing and polishing, as well as after 6, 12, and 18 days of storage in their respective solutions.

### Preparation of specimens

Thirty bar-shaped specimens, each measuring eight mm in length, five mm in width, and two mm in thickness, were created from each resin-based composite (RBC) material. The specimens were shaped to fit the spectrophotometer’s color measurement window, ensuring the consistent positioning of all specimens and equal composite coverage surrounding the window’s edges. Previous studies have used this shape for colorimetric analyses (Bozoğulları& Temizci, 2023; Mosharraf, Givehchian & Ansaripour, 2019; Shishehian et al., 2023). Table 1 summarizes the details related to the RBCs used in the study.

**Table 1:** Composite materials used in the study.

**Brand name**	**Abbreviation**	**Manufacturer**	**Material type**	**Shade**	**Composition**
Vittra APS Unique	V	FGM Dental Group, Joinville, SC, Brazil	Nano-hybrid composite	Single shade	UDMA, TEGDMA, fillers: boron-luminum–silicate glass (72–80 wt%). (Inorganic fillers load 52–60 vol.% with particle size around 200 nm), advanced photo initiator composition (APS), co-initiators, and silane. (Lot#21020)
Tetric-N-Ceram	T	Ivoclar Vivadent AG, Schaan, Liechtenstein	Nano-hybrid composite	Shade A2	DMAs (19–20 wt%), fillers: barium glass, ytterbium trifluoride, mixed oxide, and copolymers (80–81 wt%). (Total inorganic fillers load 55–57 vol.% with particle size 40–3,000 nm), additives, catalysts, stabilizers, and pigments (< 1 wt%). (Lot#Z02ZKS)

**Notes.**

UDMA urethane dimethacrylate TEGDMA triethyleneglycol dimethacrylate DMAs dimethacrylates

**Figure 2:**
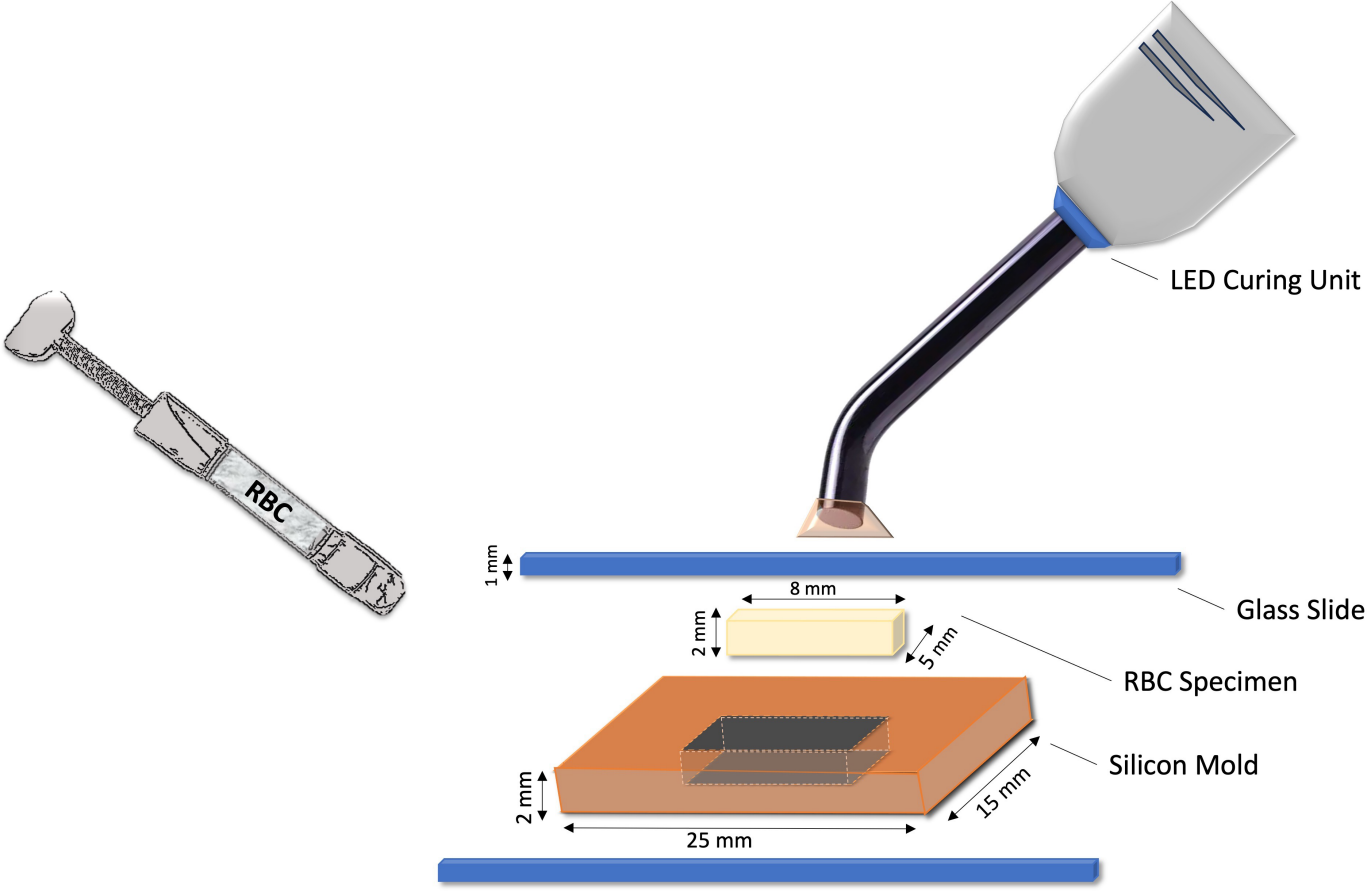
Illustration of the specimen preparation armamentarium used in the study.

A custom-made rectangular silicon mold that measured 25 mm in length, 15 mm in width, and two mm in thickness was used to fabricate the specimens. The silicon mold contained a central rectangular hole measuring eight mm × five mm × two mm for packing the RBCs. The mold was positioned on a glass slide, and then another one mm-thick glass slide was placed on top of the mold after packing the RBC into the mold in a single bulk increment. Gentle pressure was applied to the top glass slide to remove any excess material and eliminate air bubbles. Each RBC specimen was light-cured using a light emitting diode (LED) curing unit (E-Morlit, Apoza, New Taipei, Taiwan, ROC) following the manufacturer’s instructions, for 20 s from both the top and bottom surfaces. The light curing irradiance was checked frequently using a spectroradiometer to make sure it was consistent at 1,200 mW/cm^2^. The glass slides on each surface were kept in place during the polymerization procedure to ensure the standardization of the specimen’s thickness and the distance from the specimen’s surface to the curing light tip. Figure 2 illustrates the specimen preparation armamentarium used in the study.

The Super-Snap system (SHOFU Dental GmbH, Ratingen, Germany) was used to finish and polish the top and bottom surfaces of each specimen. The finishing and polishing procedures were carried out for 20 s at 10,000 rpm with abundant water cooling. Uniform specimen size was confirmed using a digital caliper (Vernier caliper; Hi-Wendy, New Taipei, Taiwan) to ensure the absence of dimensional variation. The specimens were visually inspected for defects, and any that were found to be defective were discarded. Each specimen was kept in a marked container according to its material group and immersion beverage solution subgroup.

### Preparation of beverage solutions and immersion procedures

Baseline color parameters were recorded for all specimens after the completion of the finishing and polishing procedures. Ten specimens from each material group were placed in one of the following solutions: coffee (Nescafé Classic, Nestle, Vevey, Switzerland), green tea kombucha (Zad Madina bio FOODS, Hofuf, Saudi Arabia), turmeric solution (Mehran turmeric powder, Gul M. Memon Spice Factory, Karachi, Pakistan). The coffee solution was prepared by dissolving two g of the coffee grounds in 200 mL of boiled distilled water (Hazar & Hazar, 2022). The turmeric solution was prepared by adding 0.5 g of the turmeric powder to 150 mL of distilled water (Chittem, Sajjan & Varma Kanumuri, 2017). The green tea kombucha was ready-made. The coffee, turmeric, and kombucha were allowed to reach 37 °C in an incubator before immersion. All specimens were stored in their respective solution in an incubator at 37 °C for a total of 18 days. The beverage solutions were checked daily for sufficiency and replaced every other day. All immersed specimens were kept in a dark container between readings.

### Determination of color change

The specimens were rinsed with deionized water for five minutes and then dried with tissue paper before color measurements were taken. Baseline spectrophotometric color measurements were made for all specimens according to the CIE (Commission International de L’Eclairage; Luo, 2014) L*a*b* parameters using a lab-grade desktop spectrophotometer (Color-Eye 7000A, X-Rite Incorporated, Grand Rapids, MI, USA), where L* represents lightness, with a value of 100 indicating white and zero indicating black; a* represents the red color axis (positive) and the green color axis (negative); b* represents the yellow color axis (positive) and the blue color axis (negative). A specialized jig was utilized to position the specimens against the window of the spectrophotometer at a marked location, resting on the surface to be measured. The spectrophotometric evaluation of each specimen was performed against a white background under the same light source (Illuminant D65) during all stages of the study, and by one investigator to avoid variability in color measurements. The color recording was completed for all specimens by one examiner, and the spectrophotometer was calibrated before each usage with the designated calibration block. An average of three readings for each specimen was recorded for each color parameter. After every 6 days of immersion in the beverage solutions, the specimens were removed and rinsed gently with deionized water, and then dried with absorbent paper. The color of the top surface of each specimen was recorded using the CIE L*a*b* parameters as described above. The color parameters according to CIEDE2000 were recorded, analyzing changes in lightness (ΔL00), chroma (ΔC00), and hue (ΔH00) (Gomez-Polo et al., 2016).

The color changes due to immersion in the beverage solutions (ΔE_ab_ and ΔE_00_) were determined for each time point compared to baseline measurements by applying the CIE 1976 L*a*b* (Luo, 2014) and the CIEDE2000 (Sharma, Wu & Dalal, 2005) color systems using the formulae:

ΔE_abx_ = [(ΔL_x_*)^2^ + (Δa_x_*)^2^ + (Δb_x_*)^2^]^1/2^

**Figure 3:**
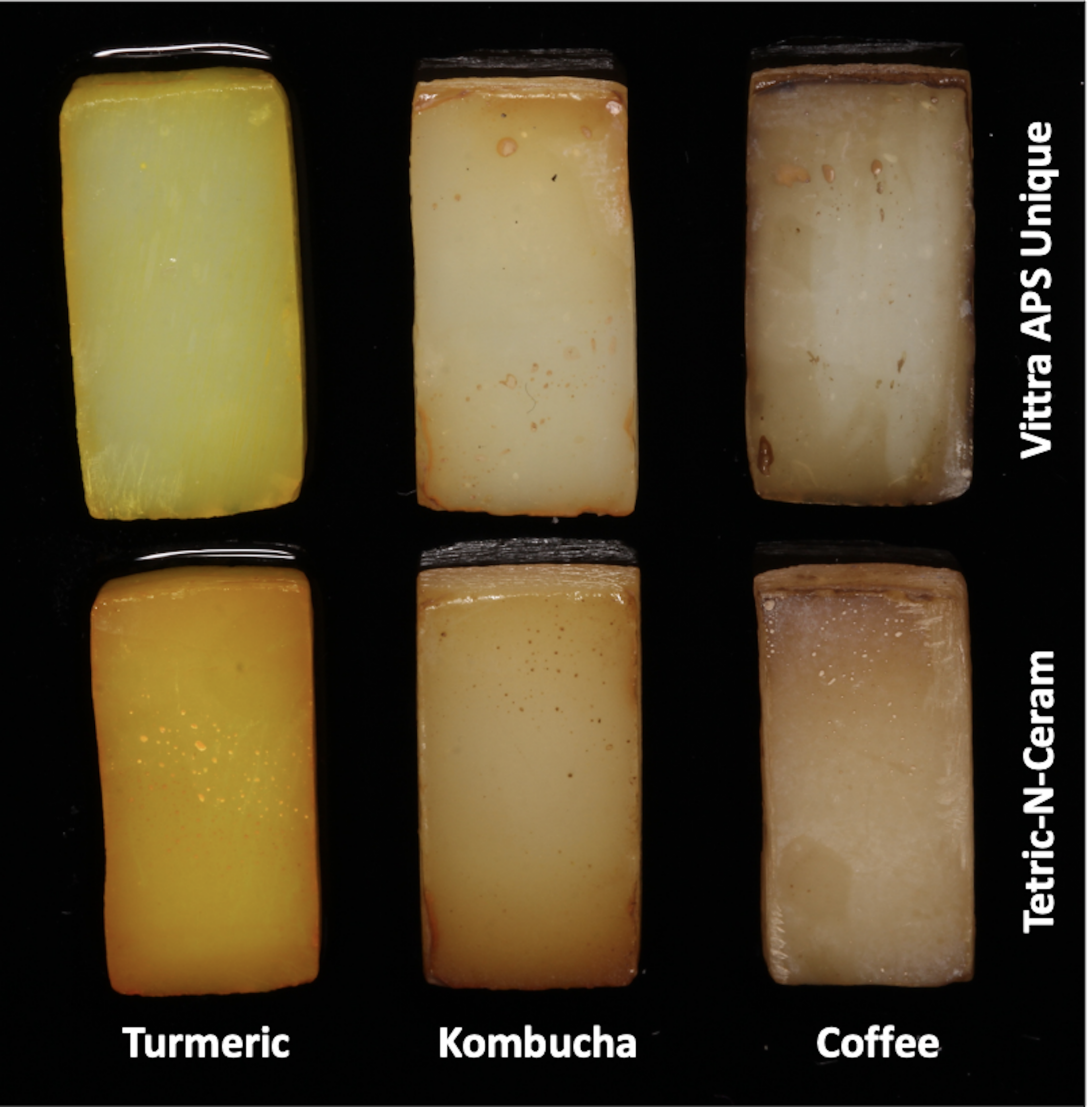
The RBC specimens at the end of the 18-day beverage solutions immersion period.

ΔE_00x_=[(ΔL′÷(k_L_×S_L_))^2^+(ΔC′÷(k_c_×S_c_))^2^+(ΔH′÷(k_H_×S_H_))^2^+ R_T_ × (ΔC′ ÷ (k_c_×S_c_))×(ΔH′÷(k_H_×S_H_))]^1/2^

where _x_ indicates the number of days of immersion in the beverage solution. The mean ΔE_ab_ and ΔE_00_ values for each subgroup were calculated. (ΔE_ab6,_ΔE_006_), (ΔE_ab12_, ΔE_0012_), and (ΔE_ab18_, ΔE_0018_) represent color changes after 6, 12, and 18 days of immersion, respectively. Figure 3 shows the beverage solutions’ staining effects on the colors of the tested RBC materials at the end of the 18-day immersion period.

### Statistical analysis

Descriptive statistics were collected on the color change in each subgroup at the three immersion time points 6, 12 and 18 days. A Shapiro–Wilk test of normality was conducted at a significance level of *p* < 0.05. Only one variable deviated slightly from normal distribution, and therefore the data were regarded as normally distributed in general, and parametric tests were applied. A repeated-measure mixed-design analysis of variance (ANOVA) was conducted with the dependent variable being color change to evaluate the interaction of effects among within and between-subject factors. Mauchly’s test of sphericity was non-significant for ΔE_ab_ (*p* > 0.05), indicating no violation of sphericity assumption, but was significant for ΔE_00_, indicating a violation of the sphericity assumption (*p* < 0.05). Therefore, Greenhouse–Geisser correction was applied to ΔE_00_ data. Multiple comparisons using the Bonferroni method were conducted to test the effect of the RBC material, beverage solution, and immersion time on the estimated marginal means of color change (ΔE_00_ and ΔE_ab_). The statistical software, SPSS Ver. 17 (IBM Corp. Released 2017. IBM SPSS Statistics for Windows, Version 25.0. IBM Corp., Armonk, NY, USA) was used at a significance level of 0.05.

## Results

Descriptive statistics for the color change (ΔE; including data of both: ΔE_ab_ and ΔE_00_) after 6, 12, and 18 days of immersion in the beverage solutions were calculated, including mean, standard deviation, confidence interval, and minimum and maximum values, as well as the clinical noticeability level of the color change mean values. These results are summarized in Table 2.

**Table 2:** Descriptive statistics of color change values for all subgroups of the tested materials in the study.

**Immersion time**	**R B C**	**Immersion beverage**	**N**	**Mean ΔE_*ab*/*x*_** ± Standard Deviation		**95% Confidence Interval for Mean ΔE_*ab*/*x*_**		**Min. ΔE_*ab*_**	**Max. ΔE_*ab*_**	**Mean ΔE_00 __*x*_** **± Standard Deviation**		**95% Confidence Interval for Mean ΔE_00 __*x*_**		**Min. ΔE_00_**	**Max. ΔE_00_**
					Lower bound	Upper bound				Lower bound	Upper bound		
6 days	V	Coffee	10	5.78^**^ ± 1.54	4.68	6.89	3.14	7.72	5.31^**^ ± 1.38	4.33	6.30	2.99	7.05
		Kambucha	10	3.87^*^ ± 1.41	2.86	4.88	1.71	5.74	3.42^*^ ± 1.16	2.60	4.25	1.57	4.93
		Turmeric	10	44.78^**^ ± 2.29	43.14	46.41	42.47	49.38	22.25^**^ ± 0.54	21.86	22.64	21.60	23.24
	T	Coffee	10	6.03^**^ ± 2.57	4.19	7.86	3.28	10.77	5.15^**^ ± 2.14	3.61	6.68	2.78	8.87
		Kambucha	10	6.30^**^ ± 1.72	5.07	7.53	4.19	9.59	4.88^*^ ± 1.05	4.12	5.63	3.46	6.71
		Turmeric	10	33.35^**^ ± 7.57	27.94	38.77	20.16	43.89	16.82^**^ ± 2.51	15.03	18.61	11.95	19.54
12 days	V	Coffee	10	10.33^**^ ± 2.65	8.44	12.23	6.46	14.45	9.15^**^ ± 2.23	7.56	10.75	5.91	12.73
		Kambucha	10	7.88^**^ ± 3.17	5.62	10.15	3.98	13.35	6.43^**^ ± 2.15	4.89	7.97	3.59	10.17
		Turmeric	10	46.82^**^ ± 4.23	43.79	49.85	37.19	50.08	22.96^**^ ± 1.16	22.12	23.79	20.43	23.90
	T	Coffee	10	9.41^**^ ± 4.58	6.13	12.69	4.22	18.51	7.85^**^ ± 3.76	5.16	10.54	3.53	15.46
		Kambucha	10	8.02^**^ ± 2.96	5.90	10.14	3.35	12.92	5.94^**^ ± 1.86	4.61	7.27	2.77	8.94
		Turmeric	10	42.52^**^ ± 6.75	37.69	47.34	29.94	50.54	19.69^**^ ± 2.14	18.16	21.22	15.97	21.93
18 days	V	Coffee	10	11.80^**^ ± 2.60	9.94	13.66	7.00	15.29	10.22^**^ ± 2.25	8.61	11.82	6.31	13.39
		Kambucha	10	8.75^**^ ± 3.33	6.36	11.13	3.47	14.17	7.11^**^ ± 2.41	5.39	8.84	3.18	11.13
		Turmeric	10	50.32^**^ ± 1.30	49.39	51.25	48.20	52.23	23.91^**^ ± 0.40	23.62	24.20	23.16	24.48
	T	Coffee	10	10.36^**^ ± 5.03	6.77	13.96	4.98	19.43	8.50^**^ ± 4.07	5.59	11.41	4.11	16.31
		Kambucha	10	9.77^**^ ± 4.69	6.42	13.13	3.61	17.46	7.11^**^ ± 2.86	5.07	9.15	3.08	11.74
		Turmeric	10	45.20^**^ ± 1.82	43.89	46.50	41.45	48.01	20.52^**^ ± 0.66	20.05	20.98	19.85	22.02

**Notes.**

RBC resin-based composite material V Vittra APS Unique (single-shade) material T Tetric-N-Ceram (multi-shade) material ΔE_*ab* *x*_ and ΔE_00__ *x*_ color change (according to the CIE Lab and CIEDE2000 formulae, respectively) after x days of immersion

∗ Indicates a clearly noticeable color change.

** Indicates an impression of two different colors.

**Figure 4:**
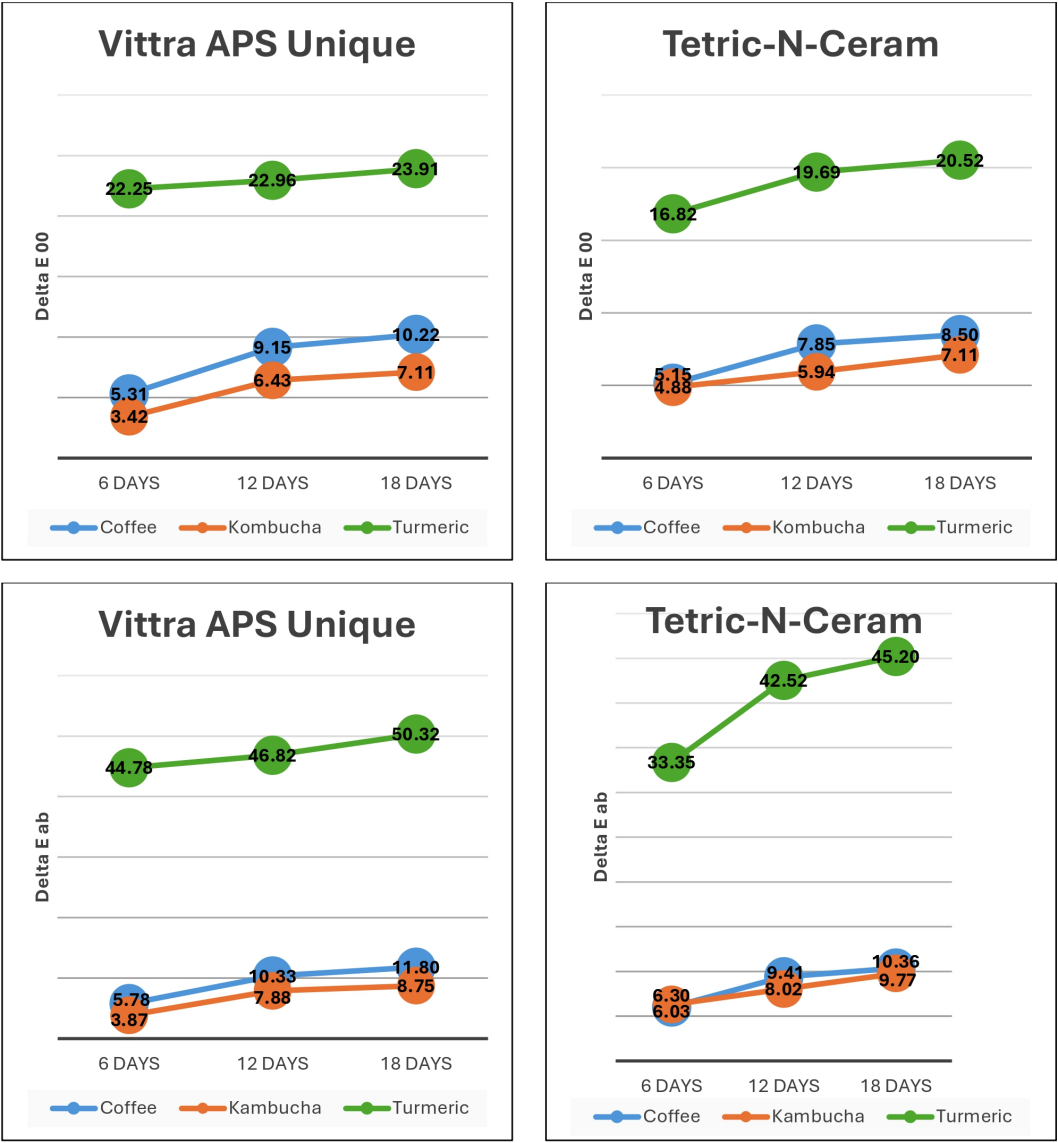
Color change (ΔE_*ab*_ and ΔE_00_) in Vittra APS Unique and Tetric-N-Ceram resin-based-composite (RBC) materials due to immersion in beverage solutions.

Both Vittra APS Unique (single-shade) and Tetric N-Ceram (multi-shade) materials exhibited the greatest color change after all immersion durations in the turmeric solution. The results from the repeated measures mixed model ANOVA tests within subjects showed that the immersion time caused a statistically significant increase in ΔE (both: ΔE_ab_ and ΔE_00_) for each RBC material (*p* < 0.001). Two-way interactions (immersion time * RBC material) had no statistically significant effect on ΔE (ΔE_ab_ *p* = 0.52, ΔE_00_ *p* = 0.78). The two-way interaction (immersion time * beverage solution) was statistically significant, with a difference in ΔE_ab_ (*p* = 0.03), but it was insignificant for ΔE_00_ (*p* = 0.17). However, the two-way interaction (RBC material * beverage solution) was statistically significant for both ΔE_ab_ and ΔE_00_ (*p* < 0.001). Figure 4 demonstrates the color change values for both tested materials in the different beverage solutions throughout the study.

The three-way interaction (immersion time * RBC material * beverage solution) also showed a statistically significant effect on ΔE (ΔE_ab_: *p* < 0.001, and ΔE_00_: *p* = 0.02). Tests of between-subjects effects on ΔE showed a statistically significant effect of RBC material (ΔE_ab_: *p* = 0.004, and ΔE_00_: *p* < 0.001) and of beverage solution (*p* < 0.001) on ΔE. The three-way interaction and between subjects effect analyses are detailed in Table 3.

**Table 3:** Three-way interaction and between-subject effects statistical results for the color change ΔE_*ab*_ and ΔE_00_.

**Three-way interaction: ΔE_*ab*_**						
		**Type III sum of squares**	**df**	**Mean square**	**F**	***P* value**
**Tests of between-subjects effects: ΔE_*ab*_**						
Intercept		72,520.34	1	72,520.34	3,171.94	<0.001^*^
Material		208.58	1	208.58	9.12	<0.01^*^
Solution		50,872.43	2	25,436.22	1,112.55	<0.001^*^
Material * solution		545.27	2	272.64	11.92	<0.001^*^
Error		1,234.61	54	22.86		
**Tests of within-subjects effects: ΔE_*ab*_**						
Time	Sphericity assumed	1,137.78	2	568.89	57.02	<0.001^*^
Time * material	Sphericity assumed	13.26	2	6.63	0.66	0.52
Time * solution	Sphericity assumed	115.23	4	28.81	2.89	0.03^*^
Time * material * solution	Sphericity assumed	159.18	4	39.80	3.99	0.005^*^
Error (Time)	Sphericity assumed	1,077.48	108	9.98		
**Three-way interaction: ΔE_00_**						
**Tests of between-subjects effects: ΔE_00_**						
Intercept		23,857.70	1	23,857.70	2,570.11	<0.001^*^
Material		113.88	1	113.88	12.27	<0.001^*^
Solution		8,249.10	2	4,124.55	444.32	<0.001^*^
Material * solution		148.28	2	74.14	7.99	<0.001^*^
Error		501.27	54	9.28		
**Tests of within-subjects effects: ΔE_00_**						
Time	Greenhouse-Geisser	339.74	2	203.65	69.95	<0.001^*^
Time * material	Greenhouse-Geisser	0.99	2	0.60	0.20	0.78
Time * solution	Greenhouse-Geisser	16.33	3	4.89	1.68	0.17
Time * material * solution	Greenhouse-Geisser	30.46	3	9.13	3.14	0.02^*^
Error (Time)	Greenhouse-Geisser	1,077.48	108	9.98		

**Notes.**

An asterisk (*) indicates an interaction with the factor.

**Figure 5:**
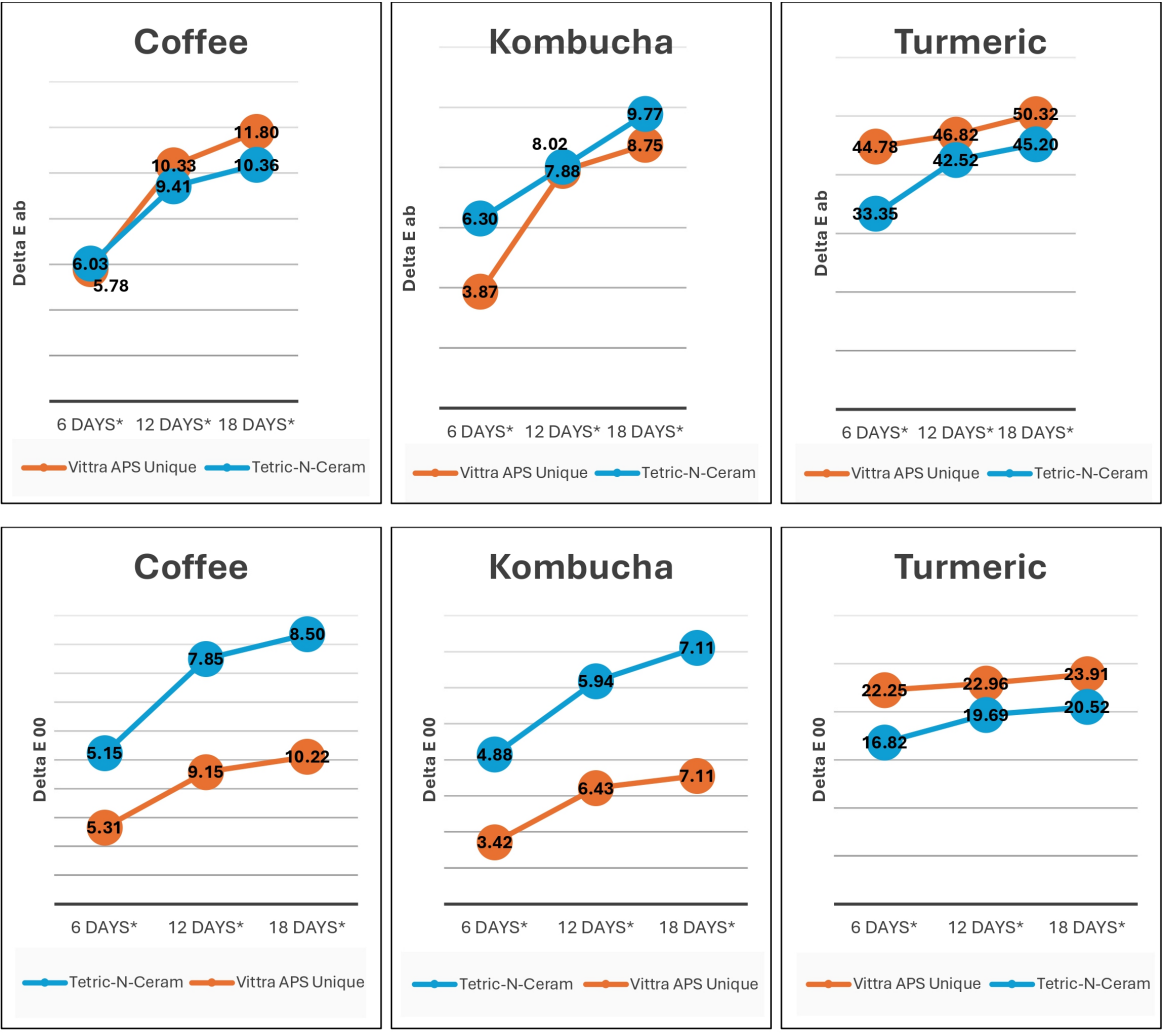
Graphs showing the statistically significant increase in color change with each increase in immersion time (* statistically significant changes (*p* < 0.001)).

Pairwise multiple comparisons of immersion times showed that each increase in immersion time resulted in a statistically significant increase in ΔE (both ΔE_ab_ and ΔE_00_) compared to the previous immersion time point (*p* < 0.001). Figure 5 demonstrates the statistically significant increase in color change from each time point to the next.

**Figure 6:**
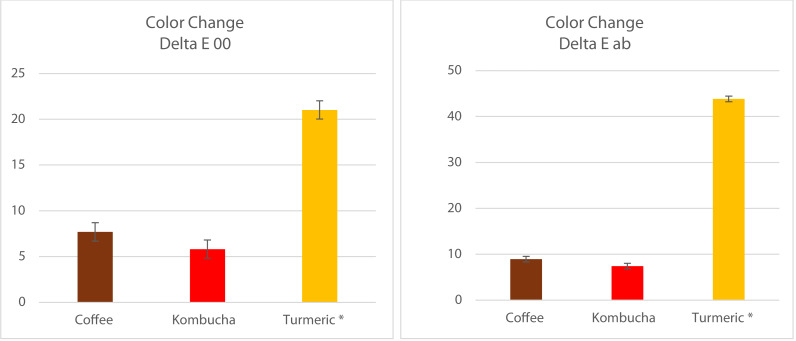
Bar graphs showing the significantly greater change in color caused by turmeric compared to both coffee and kombucha (both *p* < 0.001), which were not statistically significantly different from each other for Tetric-N-Ceram (*p* > 0.05).

The color change of Vittra APS Unique (single-shade) was statistically significantly higher than that of Tetric N-Ceram (multi-shade) according to pairwise comparisons performed using the Bonferroni method (*p* < 0.01). Additionally, pairwise multiple comparisons of beverage solutions showed that the turmeric solution produced a statistically significantly greater color change in RBC materials than both coffee and kombucha (*p* < 0.001 for both ΔE_ab_ and ΔE_00_). However, there was no statistically significant difference in the color change produced by beverage solution immersion between coffee and kombucha on color change in Tetric N-Ceram (multi-shade) RBC materials produced as a result of beverage solution immersion between coffee and kombucha (both ΔE_ab_ and ΔE_00_ *p* > 0.05). However, the coffee immersion of Vittra APS Unique (single-shade) produced a statistically significantly greater ΔE (ΔE_ab_, *p* = 0.03; ΔE_00_, *p* < 0.001) than kombucha. Figure 6 shows the total effects of immersion beverage solutions on the changes in color of the tested RBC materials.

## Discussion

While multi-shade RBCs were traditionally layered for esthetic restorations (Zafar et al., 2020), single-shade RBCs gained popularity due to their convenience and color-matching performance (Alhamdan et al., 2021). Both types were susceptible to discoloration from pigmented foods and beverages (Rohym, Tawfeek & Kamh, 2023; Tepe, Celiksoz & Yaman, 2025). The consumption of ethnic, natural, health-promoting, and caffeinated beverages, like turmeric and kombucha beverages, has increased worldwide (Gamal, Safwat & Abdou, 2022; Malhotra et al., 2011). Therefore, the current study aimed to examine the color stability of a single-shade nano-hybrid RBC with an advanced polymerization system and a nano-hybrid multi-shade RBC with Lucirin-TPO photoinitiator in response to exposure to such beverages. Shade A2 was chosen in this study because it is one of the most commonly used shades in dental practices (Abdelraouf & Habib, 2016). Nanohybrid RBCs are mostly used for esthetic, highly polished yet strong dental restorations (Maran et al., 2020). Therefore, nanohybrid RBCs were tested in the current study. The study’s results led to the rejection of both null hypotheses. The first null hypothesis of the study was that there was no significant difference in the color change of single-shade *vs.* multi-shade RBCs after exposure to the staining solutions. The second null hypothesis is that there was no significant difference in the effects of the three beverages used and/or the durations of exposure on the color stability of both tested RBCs.

In this study, the specimens were immersed in the beverage solutions for durations of 6, 12, and 18 days, simulating 6, 12, and 18 months of beverage exposure based on prior findings that 24 h of coffee immersion equated to one month of consuming 3.2 cups daily for 15 min each (Ertas et al., 2006; Yeslam & AlZahrani, 2022). The investigated immersion durations in the current study were clinically relevant as they represented the recommended recall intervals for patients with various caries risk statuses, according to the caries management by risk assessment (CAMBRA) guidelines (Featherstone et al., 2021). The specimen thickness in the current study was standardized at two mm to simulate clinical scenarios and adhere to the ISO 4049 testing standard, thereby providing a standardized comparison to previous literature, allowing uniform stain absorption, and ensuring adequate RBC polymerization (Anwar, Hussein & Riad, 2024; Bagheri, Burrow & Tyas, 2005; Bagheri et al., 2015; Ferracane, 2011; Hasanain, 2022; Müller, Rohr & Fischer, 2017; Rocha et al., 2022; ISO, 2019).

Color coordinates (L*a*b*, hue, and chroma) were measured in the current study using a lab-grade spectrophotometer according to Commission International de L’Eclairage (CIE), with a white background to minimize metamerism and compensate for chromatic shortage (Ersöz et al., 2022; Pedrosa et al., 2021). In this study, the color changes (ΔE) were determined using both the CIE L*ab and CIEDE 2000 formulas derived from the CIELAB color space, since it was reported by the International Standards Organization (ISO) and CIE that ΔE_00_ has a better ability to show color differences perceived by the human eye (Paolone et al., 2022; Sharma, Wu & Dalal, 2005; Tepe, Celiksoz & Yaman, 2025). It also correlated with perceptibility (PT_00_) and acceptability (AT_00_) thresholds better than ΔE*_ab_ (Gomez-Polo et al., 2016; Oliveira et al., 2015). This explained the detection of the significantly higher coffee-induced ΔE_00_(*p* < 0.05), when analyzing the main effect (immersion beverage), compared to kombucha, and the lack of a significant change in ΔE_ab_. Color differences were mainly perceived *via* coordinate a*, especially by women (who also considered coordinate b*), while L* played a lesser role. Only women seemed to consider coordinate b* (Gomez-Polo et al., 2016).

A previous study, categorized color changes into unnoticeable (ΔE 0–1), noticeable only by an experienced observer (ΔE 1–2), noticeable by the inexperienced observer (ΔE 2–3.5), clearly noticeable (ΔE 3.5–5), and impression of two different colors (ΔE >5) (Mokrzycki & Tatol, 2011). In the dental literature, a ΔE_00_ value of 1.8 was typically recognized as the perceptibility threshold, while 2.7 was considered the acceptability threshold (Al-khazraji & Sleibi, 2023; Paravina, Perez & Ghinea, 2019). Using the 50:50% PT_00_ and AT_00_ thresholds is also important for colorimetric assessments of teeth and restorations in clinical practice, whereby the observer’s experience and gender affected AT_00_ lightness, resulting in males and laypersons having a higher tolerance for chroma and lightness changes (Al-khazraji & Sleibi, 2023; Tejada-Casado et al., 2024). In the current study, after 6, 12, and 18 days of immersion in turmeric, kombucha, and coffee solutions, the ΔE_ab_ and ΔE_00_ exceeded the levels of clearly noticeable change (ΔE_ab_ and ΔE_00_>3.5) and both a high AT (>2.7) and PT (>1.8), suggesting a clinically relevant color change related the investigated beverages. This color change would eventually be unacceptable to patients and practitioners. The color changes in both single-shade and multi-shade RBCs due to immersion in various beverages corresponded with the results of previous studies (Çelik et al., 2011; Pedrosa et al., 2021; Tepe, Celiksoz & Yaman, 2025). Furthermore, the immersion of both tested RBCs in turmeric in the current study caused a change in color that was far beyond the levels of perception, acceptability, and impression of two different colors (>5) (ΔE_ab_ >30 and ΔE_00_>16).

Color stability referred to the ability of dental restorative materials to maintain their original shade and color. The factors affecting composite resin could be intrinsic (related to the composition of the resin) or extrinsic (related to food and drink colorants, surface finish, and polish) (Ashok & Jayalakshmi, 2017). A previous study indicated that the effective finishing and polishing of composite resin restorations could potentially reduce staining. However, some color change might still occur due to the material’s esthetic inadequacy, leading to patient dissatisfaction (Ersöz et al., 2022). The extrinsic staining of RBCs could be initially prevented by properly polishing its surface and periodically repolishing to prolong staining resistance (AlSheikh, 2019). In the current study, all surfaces of each specimen were finished and polished before immersion using a composite-appropriate finishing rotary instrument (fine and super-fine grit Super-Snap regular Pop-On disc for composites, SHOFU Dental GmbH, Ratingen, Germany) under high levels of water coolant application so as to better resemble the clinical context. The current study found that both Vittra APS Unique (single-shade) and Tetric N-Ceram materials underwent a noticeable color change when exposed to various beverages. Both materials tested in the study, Vittra APS Unique (single-shade) and Tetric N-ceram, showed matrix compositions based on dimethacrylates (DMA), including triethylene glycol dimethacrylate (TEGDMA) and urethane dimethacrylate (UDMA). TEGDMA’s high water absorption and color change susceptibility (Rohym, Tawfeek & Kamh, 2023), might have contributed to the observed color change in both RBCs when immersed in different beverage solutions in the current study.

In the current study, the single-shade RBC (Vittra APS Unique) showed a statistically significantly greater color change than the multi-shade RBC (Tetric N-Ceram) after immersion in the different solutions. This change was clearly noticeable and unacceptable for both materials. This was in agreement with the results of a recent study investigating the color stability of yet another single-shade RBC (Charisma One, Kulzer GmbH, Hanau, Germany) after immersion in coffee (Chen et al., 2024). Vittra APS Unique (single-shade) also showed a greater color change after aging in a recent study that compared it to universal multi-shade nano-filled RBCs (Fidan & Yağci, 2024). Water absorption and hydrolytic changes might cause resin material constituents to separate and affect its color stability (Ersöz et al., 2022). This was particularly true for the resin matrix, which absorbed water, causing discoloration within the material (intrinsic discoloration) (Liebermann et al., 2016). The slightly higher filler content in Tetric N-Ceram (multi-shade), as stated in Table 1, might contribute to its lower degree of color change compared to Vittra APS Unique (single-shade). In another study, Tetric N-Ceram (multi-shade) maintained its color better than the single-shade RBC tested intraorally when checked after 9 and 12 months of intraoral service (Anwar, Hussein & Riad, 2024). However, that study also reported clinically acceptable success for both types of resin-based composites (RBCs). A recent systematic review reported a comparable one-year color stability of single- and multi-shade RBCs, which is in accordance with the statements recently published by United States Public Health Service Evaluation (USPHS) and the World Dental Federation (FDI) (Leal et al., 2024). This was consistent with an *in vivo* study that found no significant difference in color stability between single-shade and multi-shade nano-hybrid RBCs. Both materials showed some color change after 6 and 12 months of use (2022). In the present study, a significant difference was found in the color change between the single-shade Vittra APS Unique (single-shade) and the multi-shade Tetric N-Ceram (multi-shade) after 6, 12, and 18 months of *in vitro* simulation. The higher color stability of Tetric N-Ceram (multi-shade), containing Lucerin diphenyl(2,4,6-trimethylbenzoyl) phosphine oxide (TPO), and the higher degree of color change in the Vittra APS Unique (single-shade), containing an advanced polymerization system (APS), could also be attributed to the different types of photoinitiators in the RBCs. RBCs containing TPO, like the investigated Tetric N-Ceram (multi shade), exhibited less yellowing compared to those containing CQ, which could be attributed to the CQ/amine significant impact on the a* and b* color coordinates (Camargo et al., 2015; Kowalska et al., 2022; Pedrosa et al., 2021). The APS used in Vittra APS Unique (single-shade) was reportedly more color stable than other CQ-containing photoinitiator systems (De Siqueira et al., 2020; Pedrosa et al., 2021), However, its small CQ content might still be the reason for the greater color change observed in the current study compared to Tetric N-Ceram (multi-shade). The efficiency of polymerization might have impacted the color stability and staining potential of the tested RBCs. This may be due to the matrix absorbing more staining beverages, deteriorating the RBC’s esthetic quality (Gonder & Fidan, 2022). Future studies comparing the degree of polymerization of Vittra APS Unique (single-shade) to that of Tetric N-Ceram (multi-shade) could provide valuable insights into how polymerization could affect the color stability of these RBCs.

In the current study, the turmeric solution produced a statistically significantly higher degree of color change in both tested RBCs than the other beverage solutions (*p* < 0.001). This color change was clearly noticeable and distinct from other colors, concerning AT and PT values (Tejada-Casado et al., 2024). This corresponded with the results of previous studies, where, turmeric solutions were found to result in a greater adverse effect on the color stability of nano-hybrid, micro-hybrid posterior, and universal micro-hybrid RBCs compared to tea and tobacco (Malhotra et al., 2011). Turmeric caused more severe color change than other food pigments (*e.g.*, paprika, saffron) and other ethnic additives (Chittem, Sajjan & Varma Kanumuri, 2017; Usha, Rao & George, 2018; Yew, Berekally & Richards, 2013). The profound effect of the turmeric solution on the color of both direct (including conventional and bioactive RBCs) and indirect dental RBCs was also confirmed in recent studies (Marchan et al., 2020; Thaliyadeth et al., 2019). In the current study, turmeric discolored both single- and multi-shade RBCs statistically significantly more noticeably than coffee, which corresponded with the results of previous studies conducted on multi-shade universal and gingiva-shaded bioactive and universal RBCs (Marchan et al., 2020; Telang et al., 2018), in which turmeric solutions produced a significantly higher color change than coffee, tea, and red wine. The yellow pigment in turmeric, combined with the aqueous media effects that were causing swelling, formation of interstitial spaces between inorganic fillers and matrix, and plasticization of the RBC’s polymeric matrix, could be the culprit behind the significantly higher color change (Ahmadizenouz et al., 2016; Checchi et al., 2024). Based on these results, Patients should be informed about the detectably unacceptable RBC discoloration caused by turmeric exposure, and preventive strategies should be explored with them.

In the current study, kombucha and coffee caused a significant color change in both tested RBCs. However, this effect was not significantly different between the two beverages in relation to the multi-shade RBC (Tetric N-Ceram (multi-shade)) (*p* > 0.05). The single-shade RBC Vittra APS Unique (single-shade) changed color significantly due to exposure to the coffee solution; more so than when exposed to kombucha (*p* < 0.05). This contradicted the results of a previous study, wherein the multi-shade nanohybrid RBC experienced the highest color change in coffee, and the single-shade RBC experienced the highest color change after immersion in milk tea (Ahmed, Jouhar & Vohra, 2022). Tea produced similar color changes in both multi-shade and single-shade RBC in another study (El-Rashidy, Abdelraouf & Habib, 2022a). Kombucha and coffee drinks have low pH levels, of around 3.5 and 5.45, respectively (Wang et al., 2022). The acidity of both drinks might increase the surface roughness of various nano-filled and micro-filled composite resins (Mert Eren et al., 2022). This roughness might lead to higher extrinsic stain attachment to the restorative surface and increase its water sorption, potentially causing eventual color changes in the single-shade and multi-shade universal composite resin materials (Ahmed, Jouhar & Vohra, 2022; Ersöz et al., 2022; Kalita et al., 2023; Rohym, Tawfeek & Kamh, 2023). Since kombucha is essentially a fermented tea solution, it could be expected that it would cause discoloration in a manner similar to milk tea (with potentially a higher pH than that of the kombucha used in the current study), which caused single-shade and multi-shade RBC discoloration due to the adsorption of polar colorants onto the surface, as reported in a previous study (Ahmed, Jouhar & Vohra, 2022). Previous studies have shown that the tannin compounds found in tea, and therefore in kombucha, could cause chemical reactions due to the presence of denaturing factors, leading to the discoloration of RBCs (Ebaya et al., 2022; Malekipour et al., 2012). Coffee-related RBC discoloration could include both the adsorption and the absorption of colorants that would have penetrated the organic RBC matrix due to its affinity for coffee’s yellow colorant (Poggio et al., 2016). Immersion time played a significant role in the changes in color of the tested RBCs. The color change increased significantly after 6, then again after 12, and then even more so after 18 days of storage in the beverage solutions. These results corresponded with a previous study wherein 30 days of water storage was found to cause a more notable color change in multi-shaded nano-filled RBCs, including Tetric N-Ceram (multi-shade) and the multi-shade variant of Vittra APS Unique (single-shade) (Pedrosa et al., 2021). A longer exposure to coffee also resulted in a greater color change in single-shade RBC restorations in a recent *in vitro* study (Khayat, 2024), which supported the results of the current study. Therefore, educating patients about the discoloration risks associated with kombucha and coffee on resin restorations could enhance treatment success and satisfaction.

This study offered valuable insights into the color stability of two commonly used types of resin-based dental restorative materials when exposed to various beverages. It highlighted the significant effects of immersion time, material, and beverage type, offering practical guidance to dental practitioners regarding treatment planning and patient education. However, certain study limitations should be taken into account. In the current study, only one type of single-shade RBC was investigated similar to previous studies with similar study designs (Barros et al., 2023b; El-Rashidy, Abdelraouf & Habib, 2022b; El-Rashidy et al., 2023; Gamal, Safwat & Abdou, 2022; Hasanain, 2022; Vejendla et al., 2023), which could limit the generalizability of the results to other single-shade RBCs. One multi-shade RBC was investigated, which may differ in composition from others on the market since it reacted differently to beverage exposure. Future studies comparing the effects of various beverages on the colors of other RBCs are recommended. Additionally, the current study investigated the color changes without investigating the effects of the immersion solutions on the surface roughness of the investigated RBCs, suggesting the need for future studies on their effects on the physical properties of these restorative materials. The study only involved an 18-day immersion period corresponding to 18 months of intra-oral use, which might not fully reflect the long-term effects on dental restorative materials. Factors such as oral hygiene practices, beverage concentration and dosage, and thermal changes were not considered, which might further limit its generalizability to real-life oral conditions. However, its results provided valuable insights into the color performance of APC system-containing single-shade resin-based composites, helping to raise dental consumer awareness. Future studies comparing different single-shade and multi-shade RBCs with identical photo initiators (such as the Vittra APS range, including the one investigated in the current study) are recommended to isolate the effects of shade technology.

## Conclusions

In conclusion, the results of this study emphasize the clinical importance of color stability in single-shade resin-based composites (RBCs), as the Vittra APS Unique (single-shade) RBC tested material in the current study, which contained an advanced polymerization system, was more susceptible to color change due to immersion in the different beverage solutions. This is significant in relation to patient satisfaction, acceptance, and the long-term performances of direct esthetic restorations.

According to the results of the current study, turmeric produced the most deleterious effects on the colors of the tested Vittra APS Unique (single-shade) and Tetric N-Ceram (multi-shade) composites. Coffee and kombucha also affected the stability of these restorative material’s color, but to a lesser extent, while coffee affected Vittra APS Unique (single-shade) single-shade RBC more than kombucha. This indicated the need for and importance of patient education regarding the impacts of beverage choices on esthetic dental restorative treatment. Future research should address the effects of more prolonged exposure to an even wider range of ethnic and non-ethnic beverages and food substances. Additionally, investigating the effects of aging, temperature, doses, and preparation of such beverages on Vittra APS Unique (single-shade) RBC compared to other Vittra APS composites and other single-shade RBCs is advised. Furthermore, the development of strategies for maintaining and monitoring esthetic restorations, and the enforcement of regular patient checkups to assess the placed restoration while educating the patients on esthetic maintenance, including offering dietary advice, could enhance their longevity of the color match achieved in clinical practice.

##  Supplemental Information

10.7717/peerj.19759/supp-1 Supplemental Information 1 Raw Data and statistical analysis of color change in both tested materials

10.7717/peerj.19759/supp-2 Supplemental Information 2 Delta E 00 statistical analysis

10.7717/peerj.19759/supp-3 Supplemental Information 3 Raw Data Registered color values.

## Additional Information and Declarations

### Data Availability

The following information was supplied regarding data availability: The raw data and statistical data of results are available in the Supplementary Files.
